# Tumor Necrosis Factor Alpha in Amyotrophic Lateral Sclerosis: Friend or Foe?

**DOI:** 10.3390/cells10030518

**Published:** 2021-03-01

**Authors:** Giulia Guidotti, Chiara Scarlata, Liliana Brambilla, Daniela Rossi

**Affiliations:** Laboratory for Research on Neurodegenerative Disorders, Istituti Clinici Scientifici Maugeri IRCCS, 27100 Pavia, Italy; g.guidotti90@gmail.com (G.G.); chiara.scarlata@icsmaugeri.it (C.S.); liliana.brambilla@icsmaugeri.it (L.B.)

**Keywords:** amyotrophic lateral sclerosis, tumor necrosis factor alpha, neuroinflammation, motor neuron degeneration, rehabilitation

## Abstract

Amyotrophic lateral sclerosis (ALS) is a fatal neurodegenerative disease characterized by a massive neuroinflammatory reaction, which plays a key role in the progression of the disease. One of the major mediators of the inflammatory response is the pleiotropic cytokine tumor necrosis factor α (TNFα), mainly released within the central nervous system (CNS) by reactive astrocytes and microglia. Increased levels of TNFα and its receptors (TNFR1 and TNFR2) have been described in plasma, serum, cerebrospinal fluid and CNS tissue from both ALS patients and transgenic animal models of disease. However, the precise role exerted by TNFα in the context of ALS is still highly controversial, since both protective and detrimental functions have been reported. These opposing actions depend on multiple factors, among which includes the type of TNFα receptor activated. In fact, TNFR2 seems to mediate a harmful role being involved in motor neuron cell death, whereas TNFR1 signaling mediates neuroprotective effects, promoting the expression and secretion of trophic factors. This suggests that a better understanding of the cytokine impact on ALS progression may enable the development of effective therapies aimed at strengthening the protective roles of TNFα and at suppressing the detrimental ones.

## 1. Introduction

Amyotrophic lateral sclerosis (ALS) is an adult-onset and fatal neurodegenerative disorder caused by degeneration of cortical, brainstem and spinal cord motor neurons. Epidemiological studies have revealed a prevalence of 3 to 5 cases per 100,000 people and an incidence of 1 to 2 cases per 100,000 people per year [1].

The early clinical manifestations of ALS can be different among individuals. In most cases (about 66%), patients show a spinal-onset suffering from fasciculations and muscular weakness of the limbs, while the remaining cases (about 33%) can present with a bulbar-onset, which is characterized by difficulty in speaking (dysarthria) and swallowing (dysphagia) [2,3,4,5]. In the later stages of the disease, both groups of patients undergo progressive atrophy of most skeletal muscles with consequent spasticity and paralysis. Although motor dysfunction is the predominant feature of ALS, up to half of the patients concurrently develop mild cognitive and behavioral decline, with a minority resulting in Frontotemporal Lobe Dementia (FTLD) associated with shorter survival [6,7]. To date, no effective cure for ALS exists and the patient death usually occurs by respiratory failure within 3–5 years from the diagnosis [3].

The care of ALS patients is multidisciplinary and includes pharmacological therapies, nutritional and respiratory support, and physical activity. The impact of rehabilitation on ALS is still controversial [8]. Some studies support the benefit of strictly monitored exercise training on the quality of life of ALS patients, because of the reduction in motor deterioration [9,10]. However, other evidence reveals negative outcomes on neuromuscular function [11]. Currently, there are only two drugs approved by the Food and Drug Administration (FDA), i.e., riluzole and edaravone, which are able to provide a modest improvement on the patient survival. Riluzole is an anti-glutamatergic agent that prolongs the lifespan of about three months in the last clinical stage of ALS [12], while edaravone is a free radical scavenger that appears to be effective in a small subset of early stage patients [13].

In the vast majority of cases, the disease appears sporadically (sALS) as a result of both environmental and genetic risk factors. However, about 5–10% of cases are classified as familial ALS (fALS); the latter exhibiting an autosomal-dominant trait of inheritance, mostly associated with causal mutations in genes that have a wide range of functions [14].

The first missense mutations were identified, in 1993, in the gene encoding the copper-zinc superoxide dismutase (SOD1) enzyme, which is responsible for redox homeostasis, and it is associated with 13% of fALS and 1% of sALS [15]. This discovery raised the possibility of generating transgenic animal models of the disease, which express mutant forms of the human SOD1 protein [16]. The phenotype of these genetically modified mice recapitulates several aspects of the human condition, being characterized by tremor, progressive muscular weakness, paralysis and premature death [17]. Since then, many other ALS-linked genes have been discovered and grouped in categories based on the cellular functions carried out by the specifically encoded protein. There are genes that alter (i) RNA homeostasis and trafficking, such as *TAR DNA binding protein 43* (*TARDBP*), *Fused in sarcoma* (*FUS*) and *C9orf72*; (ii) protein homeostasis, e.g., *Optineurin* (*OPTN*) and *TANK-binding kinase 1* (*TBK1*); and (iii) cytoskeletal dynamics, such as *Dynactin 1* (*DCTN1*). Mutations in these genes are mainly missense substitutions, except for the enormous intronic hexanucleotide repeat expansion in *C9orf72,* the most prevalent cause of fALS (about 40%) [1,18].

Downstream of each gene category, there is a number of mechanisms that operate, either individually or in combination, ultimately leading to neurodegeneration. Key mechanisms include aberrant RNA metabolism, mitochondrial dysfunction, toxic protein aggregate formation (e.g., enriched in TDP-43 and FUS), endoplasmic reticulum stress, excitotoxicity, impaired axonal transport and neuroinflammation [19]. Furthermore, structural and metabolic alterations in skeletal muscle were proposed to exacerbate the disease outcome. MicroRNAs (miRNAs) have emerged as important players in muscle proliferation and atrophy, as well as in the regulation of the inflammatory response driving the process of muscle regeneration. Recently, the group of Corrado Angelini demonstrated a significant overexpression of a group of muscle-specific (especially miR-206) and inflammatory miRNAs (especially miR-221) in muscle biopsies from ALS patients with early-onset symptoms and longer disease duration. This suggested a possible role for these miRNAs in the events modulating the disease, among which includes reinnervation or inflammation [20]. Moreover, in serum from spinal-onset patients, authors found higher levels of miR-133 and miR-206 when compared to bulbar-onset cases, which are characterized by faster disease progression. They hypothesized that miR-206 might delay the onset and progression of ALS by promoting the regeneration of the neuromuscular junction, while miR-133 may have a protective function at the synapse [21]. Finally, in skeletal muscle biopsies from patients with familial ALS, a significant upregulation of miR-206 and the inflammatory miRNAs miR-27a, miR-155 and miR-221 was observed when compared to controls [22]. Thus, it seems that multiple factors, rather than a single mechanism, may contribute to the development and progression of ALS.

The neuroinflammatory reaction is a prominent pathological feature observed in both sporadic and familial ALS as well as in other neurodegenerative conditions. This consists of a marked proliferation and activation of various glial cell types, particularly astrocytes and microglia, and infiltrating T-cells [4,23]. These observations, together with the evidence of the presence of ubiquitinated protein inclusions in glial cells, indicates that non-neuronal cells of the central nervous system (CNS) may contribute to neurodegeneration. Altogether, these findings prompt the conclusions that motor neuron cell death in ALS is a non-cell-autonomous process [24,25].

In the next sections of this review, we specifically focus on the impact of neuroinflammation on the pathogenesis and progression of ALS, highlighting the potential contribution to the disease of the pro-inflammatory cytokine tumor necrosis factor α (TNFα) in both patients and animal models.

## 2. Neuroinflammation in the Pathogenesis of ALS

Neuroinflammation is a complex and atypical inflammatory process that occurs locally in the brain parenchyma and in the spinal cord. It does not involve the peripheral immune system, with the exception of T cell infiltration from the bloodstream. This reaction can be induced by traumatic injuries, infections or chronic neurodegenerative diseases that trigger a series of biochemical and cellular responses in the CNS characterized by the activation of resident immune cells, i.e., microglia and astrocytes [2].

In ALS, these cells assume a reactive phenotype in response to aggregated protein inclusions, mitochondrial dysfunction or impaired axonal transport. They up-regulate typical molecular markers, such as CD11b and Iba1 in microglia and the intermediate filament glial fibrillary acidic protein (GFAP) in astrocytes. Furthermore, glial cells undergo morphological modifications, among which includes the enlargement of their cell body and the thickening of their processes [26], as well as alterations in the pattern of gene expression. These changes are associated with the overproduction of pro-inflammatory cytokines together with other neurotoxic and neuroprotective molecules, both in patients and animal models of ALS [27,28,29,30]. Once microglia are activated, the infiltration of T-cells into the CNS starts and triggers the microglial acquisition of specific properties as antigen-presenting cells [31].

When transient, these changes in glial cells are meant to protect the CNS from injury. However, in ALS and other neurodegenerative conditions, neuroinflammation is a chronic event that can lead to dysregulation of the neuronal microenvironment, and thus contribute to neurodegeneration. The first demonstration of the existence of a cause-effect relationship between glial cell activity and loss of motor neurons in ALS came from early studies performed in the mid 1990s. These investigations documented a defect in the astrocyte-specific glutamate transporter GLT1/EAAT2 in motor cortex and spinal cord from both patients and mutant SOD1 (mSOD1) mouse models [32,33]. In physiological conditions, this transporter is involved in the maintenance of extracellular glutamate concentrations below excitotoxic levels. Thus, its deficit may cause abnormal increases in the extracellular levels of the amino acid and contribute to excitotoxic motor neuron cell death. Ten years later, further studies in SOD1^G37R^ transgenic mice showed that the selective ablation of mSOD1 either in microglia or in astrocytes by means of the Cre/loxP recombination system positively modulates the phenotype, slowing down the later phase of disease progression [25,34]. A similar study on SOD1^G85R^ mice, conducted by the same technology and deleting mSOD1 selectively in astrocytes, delayed the onset of ALS and prolonged the early phase of disease progression without affecting the late phase. These apparent discrepancies between these two investigations may be explained by the different SOD1 mutations expressed in the experimental paradigms [35]. Subsequently, the transplantation of astrocyte precursors harboring the mutant SOD1^G93A^ protein into the cervical spinal cord of wild-type rodents revealed that mutant astrocytes alone can induce motor neuron cell death [36]. Conversely, the transplantation of mouse wild-type glial restricted precursors (GRPs) or human induced pluripotent stem cell (iPSC)-derived glial rich neural progenitors (GRNPs) into the spinal cord of mSOD1 mice led to their efficient differentiation into healthy astrocytes and prolonged the animal survival [37,38]. More recently, it has been shown that astrocytes derived from sALS patients and transplanted into mice can cause motor neuron degeneration, neurofilament disorganization, and ubiquitin inclusions [39]. Altogether, these findings confirm a mutual interaction between astrocytes, microglia and motor neurons in the context of ALS.

Several lines of evidence indicate that neuroinflammation is present in human post-mortem samples, in ALS patients in vivo, and in rodent models of disease, highlighting the importance of reactive glial cells in motor neuron cell death. More specifically, initial analyses of autoptic tissues obtained from sALS and fALS revealed the presence of microgliosis and T-cell infiltrates in motor cortex, motor nuclei of the brain stem, corticospinal tract and the ventral horn of the spinal cord [40,41,42]. Widespread astrocyte reactivity was also found in both cortical grey matter and subcortical white matter [43,44], as well as in the ventral and dorsal horns of the spinal cord [45]. Yet, these observations of gliosis presented the peculiar disadvantage of arising from ALS post-mortem tissues, i.e., at the final stage of the disease. Analyses of earlier phases were made possible by the advent of new neuroimaging technologies, such as positron emission tomography (PET). In living ALS patients, PET revealed massive microgliosis in several brain areas, among which are primary motor, supplementary motor and temporal cortex. Besides this, a significant correlation between the intensity of microglial activation and the severity of upper motor neuron deficits was demonstrated [46,47].

A similar pattern of gliosis was described also in transgenic ALS mouse models, though the onset of activation was shown to vary between transgenic rodent strains. Several studies performed in mSOD1 mice show that microglia and astrocytes become reactive in the ventral spinal horns before motor neuron loss and the onset of symptoms, and the process enhances until the endstage of the disease [48,49,50,51,52]. This evidence confirms that gliosis and neuroinflammation are not only secondary events or consequences of motor neuron loss, but they can play key roles in neurodegeneration and disease progression.

Once activated, microglia can display two different phenotypes: (i) M1, which is detrimental for motor neurons because of the ability of these cells to secrete cytotoxic molecules, and (ii) M2, which is characterized by the microglial production of anti-inflammatory cytokines and neurotrophic factors, thereby resulting beneficial for motor neurons. With regard to this point, recent studies carried out by using mSOD1 transgenic mice have provided evidence that during the early phase of the disease, M2 markers are up-regulated in the spinal cord, thus indicating that reactive microglia display an M2 phenotype that promotes repair and regeneration. Conversely, M1 microglia predominate during the late phase of ALS, secreting pro-inflammatory cytokines that exacerbate neuroinflammation and degeneration [53,54]. Thus, it emerges that, in the context of neurodegeneration, microgliosis can exert either neuroprotective or neurotoxic functions, depending on the phenotype acquired by the cells during the different stages of the disease.

In ALS, microglial cells closely interact with T-cells to mediate the inflammatory response. During the early phase of the disease, mSOD1 mice were reported to overexpress CD4^+^ T lymphocytes, particularly the CD4^+^CD25^High^FoxP3^+^ regulatory T-lymphocyte (Treg) subtype. The latter were shown to suppress the inflammatory process occurring in lumbar and cervical spinal cord and to support the activation of M2 microglia, thereby leading to a deceleration of disease progression [53,55,56]. In fact, Tregs inhibit detrimental immune reactions, down-regulating the production of pro-inflammatory cytokines and suppressing the activation of CD4^+^CD25^−^ effector T-lymphocytes (Teffs) through the release of anti-inflammatory molecules, such as interleukin-10 (IL-10) and transforming growth factor-β (TGF-β). On the other hand, in the later stages of ALS, mSOD1 mice were shown to be characterized by a reduced number of neuroprotective lymphocytes, with the predominance of Teffs and the conversion of microglia to the M1 phenotype [53,55]. In a prospective study on ALS patients, it was reported that an early reduction of the Treg transcription factor FoxP3 was predictive of rapid disease progression and reduced survival [57]. Another recent study documented that circulating Tregs in ALS patients were less effective in suppressing Teffs proliferation when compared to healthy controls. Interestingly, this dysfunction was more marked in rapidly progressing patients [58]. Related to this point, it is important to mention that TNFα, one of the main pro-inflammatory cytokines released by reactive glia, can either promote Tregs proliferation or inhibit their suppressive function by downregulating FoxP3 expression. This latter effect probably depends on synergy with other inflammatory factors, such as IL-6, during the later phase of the disease [59,60].

The specific functions exerted by reactive astrocytes in ALS are not well defined, and both beneficial and harmful activities have been attributed to these cells. Reactive astrocytes typically show increased immunoreactivity for GFAP and the calcium binding protein S100β. Furthermore, they exhibit the over-expression of both inflammatory and neurotoxic factors, such as inducible nitric oxide synthase (iNOS), cytokines and the cyclooxygenase-2 (COX-2) enzyme, leading to the generation of prostaglandins [61], as well as anti-inflammatory molecules and neurotrophic factors [62]. Surprisingly, a recent study showed that the anti-inflammatory cytokine TGF-β1, usually involved in immune system homeostasis and neuroprotection, was up-regulated in astrocytes of ALS patients and mice and accelerated disease progression. More specifically, Endo et al. demonstrated that astrocyte-specific over-production of TGF-β1 in mSOD1 mice did not prolong their survival through its anti-inflammatory action, but negatively affected the mouse lifespan by inhibiting the neuroprotective inflammatory responses coordinated by microglia and T-cells [63,64]. Based on this, it is reasonable to hypothesize that, in pathological conditions, the deleterious or beneficial effects of a cytokine is strictly related to its expression levels, which might trigger a shift in its activity.

In conclusion, this evidence, emerging from both transgenic mice and ALS patients, suggests that neuroinflammation is not only a secondary reaction to motor neuron injury, but may contribute to the early stages of the disease by exerting a dual function, i.e., contributing to neuroprotection and/or leading to neurotoxicity. During the early phase of the disease, glia and T-cells develop a neuroprotective compensatory response, trying to repair motor neuron damage via the production of both anti-inflammatory cytokines, such as IL-4 and IL-10, as well as growth factors, including insulin-like growth factor 1 (IGF-1), glial cell-line derived neurotrophic factor (GDNF) and brain-derived neurotrophic factor (BDNF). The increased damage to motor neurons that accompanies the progression of ALS leads to a transition from a neuroprotective response to an injurious action of neuroinflammation associated to an up-regulation of several chemokines, reactive oxygen species and pro-inflammatory cytokines, such as interferon-γ (IFN-γ), IL-1β, IL-6 and TNFα [28,29,65,66]. Although trophic factors are continuously produced, they cannot revert the ongoing process of motor neuron degeneration. 

Additional regulatory inputs to the neuroinflammatory reaction can be provided by physical exercise. It is widely acknowledged that this latter can be employed to improve remodeling of neuronal circuits and to promote functional recovery in pathological conditions, but little is known about its impact on glial cells. Yet, recent observations suggest that physical activity can significantly contribute to reduce reactive astrocytosis [67,68,69,70], to regulate microglial activation [71] and to alleviate the neuroinflammatory response in various animal models of injury and disease [72,73,74]. This supports the view that rehabilitative training can be used to regulate the inflammatory response of the nervous system.

The contribution of TNFα to the neurodegenerative process in ALS is still controversial. Both neurotoxic and neuroprotective roles have been described for this cytokine in the disease progression and the occurrence of one or the other event may be determined by its expression levels, duration of action, type of activated receptor, as well as by the state of the surrounding microenvironment.

## 3. Tumor Necrosis Factor Alpha

TNFα is a pleiotropic cytokine that is involved in the regulation of a wide spectrum of physiological and pathological processes. It is synthesized as a 26 kDa type II transmembrane protein (mTNFα) inserted into the membrane as a stable homotrimer. Its extracellular domain is cleaved by the metalloprotease TNFα-converting enzyme (TACE), producing a 17 kDa soluble monomeric form of the cytokine (sTNFα) that can subsequently generate a 51 kDa trimetric form. Both mTNFα and the trimetric sTNFα are biologically active and can be released in the CNS, mainly by glial cells but also by some populations of neurons and infiltrating T-cells [75,76].

TNFα mediates its effects through the activation of two different surface receptors (TNFR), TNF receptor 1 (TNFR1) and TNF receptor 2 (TNFR2), which differ in their expression pattern, binding affinity for ligand, cytoplasmic tail structure and downstream signaling pathways. TNFR1 contains a cytoplasmic death domain (DD), is expressed in almost all CNS cell types and can be preferentially activated by sTNFα, whereas TNFR2 is mainly expressed by microglia and endothelial cells and is the preferred target of mTNFα [75]. The extracellular domains of TNFR1 and TNFR2 can be also released from the cell surface into the bloodstream. In this soluble form, they can still bind TNFα, preventing its biological effects on cells [77,78].

Both receptors mediate signals through the recruitment of adaptor proteins, depending on which, as TNFα elicits different cellular responses. Briefly, the binding of TNFα to TNFR1 leads to the formation of two different complexes that require the initial interaction between the DD of the receptor and the TNFR-associated death domain protein (TRADD). TRADD allows the recruitment of other adaptor proteins. These include (i) receptor-interacting protein 1 (RIP1) and TNFR-associated factor 2 (TRAF2), which form the Complex I; and (ii) Fas-associated death domain protein (FADD), which recruits caspase-8 and forms the Complex II, thereby triggering cell death and anti-inflammatory processes [76,79,80]. Binding of TNFα to TNFR2 results in direct recruitment of TRAF2, and the following interaction with TRAF1. This latter complex is analogue to Complex I of TNFR1, and both trigger signals leading to the activation of various transcription factors, i.e., (i) activator protein-1 (AP-1), through the phosphorylation of p38 MAP kinase (p38MAPK) and c-jun N-terminal kinase (JNK), as well as (ii) nuclear factor kappa B (NFκB), through the I-κB kinase (IKK) complex. These pathways induce the expression of genes involved in anti-apoptotic and pro-inflammatory events.

TNFα and NFκB have been shown to participate in positive feedback loops in which NFκB can regulate TNFα transcription, while autocrine and paracrine TNFα can, in turn, trigger NFκB activation [81]. NFκB was reported to be up-regulated in spinal cord astrocytes and microglia from both ALS patients and mSOD1 mice [82,83,84]. Initial investigations showed that inhibition of NFkB selectively in astrocytes did not increase motor function or survival in SOD1^G93A^ ALS mice [84,85], while deletion of NFkB signaling in microglia extended the mouse lifespan by reducing the release of pro-inflammatory markers produced by M1 microglia [84]. Yet, in a recent study, Alami and colleagues revealed that the relevance of NFkB activation in mSOD1 mouse astrocytes is time-dependent because, during the pre-symptomatic stage of the disease, the transcription factor triggers microglial proliferation with neuroprotective effects on motor neurons; whereas in the later stages of ALS, NFkB enhances pro-inflammatory microglia activation, accelerating disease progression and resulting in a shorter mouse survival [86]. This finding supports the idea that neuroinflammation can assume a neuroprotective role during the early phase of the disease, while it becomes injurious in the later phase.

Two independent groups of researchers investigated the correlation existing between TNFα and the SOD1 gene and protein. Firstly, Afonso and colleagues studied the molecular and cellular mechanisms involved in TNFα-dependent regulation of SOD1 gene expression in cultured U937 cells. They reported that TNFα down-regulates SOD1 transcripts, protein, and promoter activity involving the JNK/AP-1 pathway [87]. More recently, in the early 2020, the group of Christine Vande Velde, identified the ubiquitin ligase TNF receptor-associated factor 6 (TRAF6), a downstream actor of multiple TNFR superfamily members, as a novel interactor of mitochondria-associated misfolded SOD1. In cellulo, they demonstrated that this interaction takes place only with mutant, but not wild-type SOD1, through the C-terminus of TRAF6. As shown in other neurodegenerative diseases, TRAF6 can stimulate the polyubiquitination and/or aggregation of mutant SOD1, negatively affecting its turnover and leading to its cellular accumulation. Since TRAF6 is expressed in the cell types harboring misfolded SOD1 in vivo, such as neurons and astrocytes, this phenomenon becomes relevant in the case of misfolded SOD1-linked ALS [88].

Growing evidence indicates that constitutive levels of TNFα are expressed in the healthy brain, both in rodents and in humans [89,90]. In these conditions, this cytokine exerts regulatory functions on important physiological processes, such as learning and memory, sleep, neurogenesis and synaptic transmission and plasticity [91,92,93]. Concerning this latter function, it is important to mention that astroglial TNFα was shown to regulate synaptic strength through mechanisms of synaptic scaling. In particular, TNFα strengthens excitatory synaptic transmission increasing the expression of calcium-permeable α-amino-3-hydroxy-5-methyl-4-isoxazolepropionic acid (AMPA) receptors, while it decreases inhibitory synaptic strength through the endocytosis of γ-aminobutyric acid type A (GABA_A_) receptors [94,95,96,97]. Besides this, basal levels of the cytokine were shown to exert neuroprotective effects by promoting the expression of various trophic factors in the CNS via TNFR1 signaling [98]. Thus, TNFR1 not only appears to mediate cell death pathways, but also neuroprotective ones.

## 4. TNFα Role in ALS: Evidence from Patients and Animal Models 

The exact role TNFα plays in ALS pathogenesis and progression is still highly controversial due to the pleiotropic nature of this cytokine.

Several studies have demonstrated a dysregulation of the TNFα/TNFR system in both ALS patients and animal models of disease, even if there are conflicting opinions as to whether the cytokine exerts neuroprotective or neurotoxic effects.

### 4.1. ALS Patients

The levels of both TNFα and its soluble receptors (sTNFRs) were reported to be significantly higher in plasma and cerebrospinal fluid (CSF) of ALS patients when compared to healthy controls. However, in multiple studies, the extent of activation of the TNFα/TNFR system did not correlate with disease duration, severity or weight loss [28,29,99,100,101,102]. Poloni and colleagues showed that, even after dividing patients into two subgroups, characterized by high and low TNFα levels, differences in terms of clinical parameters of the disease were not noticeable [28]. Conversely, Babu et al. reported that high concentrations of TNFα in serum correlated with the progression of the disease in sALS patients [103]. Further, Fukazawa and co-workers found in the skin of sALS patients numerous cells with increased TNFα immunoreactivity that became more intense with disease progression. This increment may be ascribed to reduced degradation, enhanced synthesis and/or increased binding of circulating TNFα to skin components [104].

**Figure 1:**
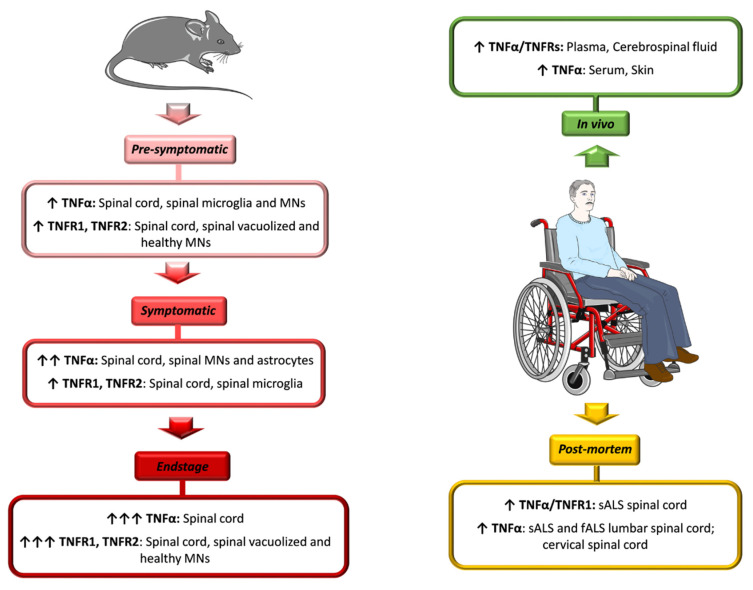
Schematic representation of the levels of TNFα and its receptors during amyotrophic lateral sclerosis (ALS) progression. Since the existence of a neuroinflammatory process is well known in neurodegenerative diseases, including ALS, numerous investigations have focused on studying the trend of concentrations of the pro-inflammatory cytokine TNFα and its receptors in ALS transgenic mouse models (↑ indicates increases in TNFα, TNFR1 or TNFR2 concentrations in the indicated tissues/cells), identifying a linear relationship between TNFα levels and stage of disease progression. Further observations were also made on living and post-mortem patients.

Intense TNFα immunoreactivity was also detected in motor neurons of lumbar spinal cord sections from sALS and a fALS patient with SOD1 mutation [105]. A more recent investigation, conducted by using next generation RNA sequencing analysis, identified a significantly elevated inflammatory process in post-mortem cervical spinal cord from ALS patients, with TNFα as main regulatory molecule [106]. Finally, our group detected significantly higher levels of TNFα and TNFR1 in autoptic sALS spinal cords when compared with healthy controls [62] (Figure 1).

Although some evidence identifies the activation of the TNFα/TNFR system as a non-specific response to degeneration of motor neurons, other studies show a correlation between the increment in the cytokine levels and the progression of disease, suggesting an important contribution of the TNFα/TNFR system to the pathogenesis of ALS. 

### 4.2. ALS Animal Models

High levels of TNFα and its receptors were also detected in the spinal cord of mSOD1 mice, and positively correlated with disease progression (Figure 1).

Hensley et al. displayed a three-fold increase in the amount of TNFα in the spinal cord of SOD1^G93A^ mice at the symptomatic stage of ALS when compared to age-matched non-transgenic mice. This was associated to a strong up-regulation of TNFR1 at the late pre-symptomatic phase [65,107].

Consistent with this, our group demonstrated that, in the spinal cord of the same mouse model, there was a concurrent increment in TNFα and TNFR1 transcripts at the age of disease onset, probably as a reaction to the initial manifestations of ALS. This increment reached the peak at the later symptomatic stage of the disease. Besides this, in SOD1^G93A^;tnfr1^−/−^ double mutant mice, the rise in TNFα transcript levels was almost completely abolished [62]. This effect was explained by the existence of an autoregulatory loop through which TNFα can modulate its own expression via TNFR1, thereby exacerbating the neuroinflammatory response.

Using cDNA microarray analysis, Yoshihara and collaborators reported a significantly up-regulation of inflammation-related genes, among which includes the TNFα gene, in SOD1^G93A^ mouse lumbar spinal cord. They showed a five-fold increase at 11 weeks of age (pre-symptomatic stage), which further enhanced at 17 weeks (endstage), when compared to age-matched non-transgenic mice. Immunohistochemical analyses revealed that TNFα was localized mainly in reactive microglia and motor neurons at 11 weeks, suggesting once again the existence of a neuron-glial cross-talk during the inflammatory process typical of neurodegenerative mechanisms. In addition, the up-regulation of TNFα was accompanied by the over-expression of apoptosis-related genes, such as caspase-1, suggesting a correlation between the inflammatory reaction mediated by the cytokine and the apoptotic pathway involved in motor neuron cell death [108].

In spinal motor neurons of SOD1^G93A^ mice at the pre-symptomatic stage, sustained activation of the p38MAPK pathway, involved in neurodegenerative mechanisms, was also reported. This activation became evident in reactive astrocytes and microglia during the progression of the disease. Examining receptors potentially involved in the phosphorylation cascade of p38MAPK, the authors detected, before the symptom onset and up to the endstage, a specific over-expression of TNFR1 and TNFR2, both in vacuolized motor neurons and in healthy neurons. This evidence confirmed that the activation of the signaling cascade leading to MAPK activation was mediated by TNFα. Since this phenomenon occurred early during the development of disease, it was considered a pathogenic mechanism. Furthermore, the authors found an up-regulation of TNFα receptors also in microglia, during the symptomatic phase. This suggests that microglia may trigger a self-sustaining mechanism of activation of the p38MAPK pathway, which may contribute to a rapid progression of the disease [109]. Kiaei et al. investigated the temporal pattern of TNFα immunoreactivity in the lumbar spinal cord of SOD1^G93A^ mice. They showed moderate TNFα staining in motor neurons of the ventral horn before the onset of symptoms. Conversely, at the symptomatic stage, immunoreactivity became more intense and occurred also in the astrocytes [105] (Figure 1).

All these data, along with those derived from ALS patients, confirm that TNFα and its receptors are already up-regulated in blood, CSF and/or in the affected tissues before symptom onset or at early symptomatic stage. Then, levels further increase during the progression of the disease. In view of these considerations, other studies have explored the effects of TNFα signaling dysregulation in ALS pathogenesis and progression.

It was specifically reported that the neuroinflammatory response, and in particular TNFα over-expression, might exacerbate glutamate-mediated excitotoxicity, a key event for motor neuron cell death in ALS. In this regard, Tolosa and collaborators, using rat spinal cord organotypic cultures, demonstrated that the exposure to high concentrations of exogenous TNFα potentiated experimentally induced chronic glutamate excitotoxicity, and consequently loss of neurons. They reported both a down-regulation of the astroglial glutamate transporter-1/excitatory amino acid transporter 2 (GLT1/EAAT2), which resulted in increased levels of extracellular glutamate, as well as an induction of oxidative stress, through a mechanism involving the activation of the NFκB signaling pathway. These results further confirm the hypothesis that TNFα plays an important role in ALS [110].

With regard to the detrimental actions triggered by TNFα, the group of Davide Trotti revealed the involvement of the cytokine also in mediating pathological blood-brain barrier (BBB) changes that might lead to pharmacoresistance over the course of ALS progression. The BBB is a dynamic and protective interface that separates the cerebral vasculature from the brain parenchyma, protecting the CNS from circulating pathogens and toxins, regulating the transport of essential molecules and limiting the passage of most drugs after systemic administration. Numerous studies have established that the BBB is damaged in ALS, a phenotype common to sALS and fALS patients, as well as to mSOD1 transgenic mice [111,112,113]. To possibly counteract this impairment, the multidrug resistance transporter P-Glycoprotein (P-gp) becomes over-expressed in the spinal cord of SOD1^G93A^ mice and increases its activity specifically in capillary endothelial cells [114,115]. Since this protein is deputed to expel substances out of cells, and because riluzole is a P-gp substrate [116], Qosa et al. suggested that riluzole-limited efficacy could be due, at least in part, to the upregulation of P-gp. Using both in vitro mouse and human models of BBB, the authors found that different ALS-linked mutations triggered distinct mechanisms that converged to increase P-gp expression. In particular, they showed that mutant SOD1-expressing astrocytes promoted an up-regulation of P-gp in endothelial cells via NFκB activation by oxidative stress. On the other hand, astrocytes expressing the mutant FUS protein (mFUS astrocytes) induced an inflammatory response by increasing TNFα secretion and stimulation of TNFR1, which in turn enhanced NFκB nuclear translocation, resulting in P-gp up-regulation [115].

**Figure 2:**
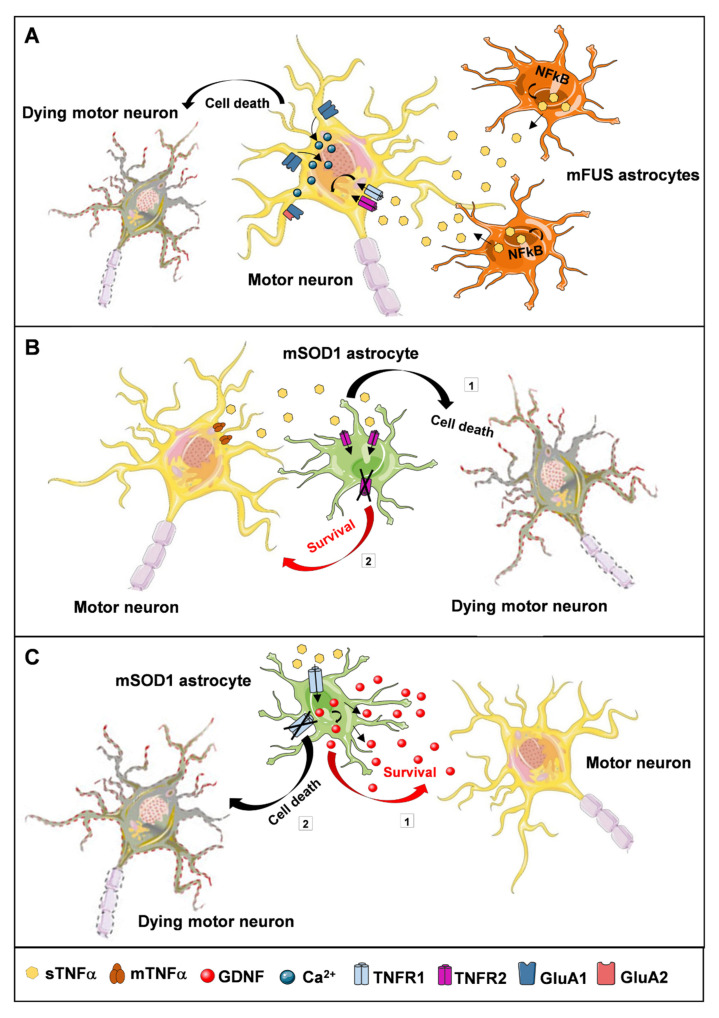
Representative drawing of the TNFα-dependent interactions occurring between astrocytes and motor neurons in ALS. The ways in which astrocytes can affect the conditions of motor neurons via TNFα signaling are numerous and different, depending on the receptors that are activated. (**A**) Mutant fused in sarcoma (FUS) astrocytes release a large amount of TNFα, produced by NFkB activation. This leads to an alteration in the expression levels of the AMPA receptor subunits GluA1 and GluA2, which causes an increase in intracellular calcium concentration with consequent death of motor neurons. (**B**) The stimulation of TNFR2 by TNFα shows a negative role on motor neurons, leading to their degeneration (1). Consistent with this, inhibition or ablation of TNFR2 significantly reduces neuronal cell loss (2). (**C**) Conversely, the stimulation of TNFR1 by exogenous TNFα can induce the expression and secretion of GDNF by mutant SOD1 astrocytes (1), favoring the survival of motor neurons. In keeping with this, TNFR1 deletion accelerates motor neuron degeneration (2).

Using primary mFUS astrocytes co-cultured with primary motor neurons, the same group of researchers found that mutant astrocytes release also a large amount of toxic factors. sTNFα was identified as one of the most abundantly secreted. The latter acted as a paracrine mediator driving damaging actions on neighboring motor neurons. More specifically, the authors observed reduced neurite length and significant neuronal loss that were rescued by targeting sTNFα with a neutralizing antibody. In addition, inhibiting the activation of NFκB reduced TNFα secretion by mFUS astrocytes, preventing motor neuron degeneration and suggesting once again the existence of a positive feedback loop that correlates NFκB and TNFα. Further, mFUS astrocytes triggered changes in the expression levels of the AMPA receptor subunits GluA1 and GluA2. In particular, they found increased GluA1 and decreased GluA2 expression in dendrites of motor neurons, which resulted in increased calcium influx leading to excitotoxic damage and cell death. Even in this case, astrocytic TNFα was the paracrine mediator responsible for AMPA receptor changes, which were prevented by TNFα neutralization [117] (Figure 2A).

A considerable inflammatory response, characterized by high levels of TNFα, was reported also in hindlimb muscles of symptomatic and endstage SOD1^G93A^ rats [118]. Because TNFα is known to play a role in muscle proteolysis [119], it reasonable to hypothesize that elevated concentrations of the cytokine in skeletal muscles might contribute to muscle wasting in both SOD1 rodents and, possibly, ALS patients, thereby influencing disease progression.

In view of this evidence, one can postulate that targeting TNFα signaling may have a therapeutic potential for the treatment of ALS. In this regard, Kiaei and colleagues tested in SOD1^G93A^ mice the neuroprotective effect of thalidomide and its analogue lenalidomide, two pharmacological agents that reduce, among others, TNFα production by destabilizing its mRNA. In particular, pre-symptomatic oral administration of both compounds resulted to attenuate weight loss, to enhance motor performance, and to decrease motor neuron cell death in the lumbar spinal cord. Besides this, the treatment delayed disease onset and slowed down disease progression, resulting in a significant increase in the mouse lifespan. All these effects were associated with a marked reduction in TNFα immunoreactivity in both motor neurons and glial cells in the lumbar spinal cord when compared to untreated mice [105]. Based on these promising preclinical data, a single-center phase II clinical trial (ClinicalTrials.gov Identifier: NCT00140452 - Study Start date: 2005) was conducted to evaluate the safety and efficacy of thalidomide in sALS and fALS patients. However, it did not effectively modulate disease progression, while it caused negative effects on survival and pulmonary function. In addition, treatment was associated with significant adverse events, including deep venous thrombosis and peripheral neuropathy [120]. This negative response, compared to that obtained in mSOD1 mice, may be ascribed to the different stage of disease in which mice and patients received the pharmacological compounds. In fact, while the mouse treatment was made before symptom onset, patients were treated after the diagnosis, when motor neurons are already significantly compromised.

Despite several studies reporting high levels of TNFα in both ALS patients and animal models, supporting its involvement in ALS pathogenesis and encouraging the development of therapies targeting TNFα, other investigations gave apparently different results and questioned the actual deleterious contribution of the cytokine to motor neuron degeneration. With regard to this point, Gowing and collaborators showed that constitutive ablation of the gene encoding TNFα did not affect survival and axonal degeneration of SOD1^G93A^ or SOD1^G37R^ mice. Moreover, no differences in terms of microgliosis or astrocytosis were detected in the lumbar ventral horns of mSOD1 mice harboring or lacking TNFα. Probably this approach, which totally abolished the cytokine signaling during both development and adult life, activated compensatory processes by other pathways. In keeping with this, a significant increase in transcripts coding for IL-1β, a pro-inflammatory cytokine that partially shares the same intracellular signaling with TNFα, was reported in spinal cords from SOD1^G93A^ mice lacking TNFα at the early symptomatic stage [121].

Furthermore, recent evidence shows that TNFR1 and TNFR2 may act in opposite directions in the context of ALS. In other words, TNFα may exert either protective or deleterious effects depending on which type of receptor is activated [62,122]. On the one hand, it has been postulated that TNFR2 could play a detrimental role, inducing selective motor neuron cell death. Studying astrocyte-neuron co-cultures from SOD1^G93A^ mice, the group of Caterina Bendotti showed both an up-regulation of mTNFα in motor neurons as well as an over-expression of TNFR2 in astroglial cells that resulted in increased motor neuron degeneration. The inhibition of TNFR2 by a neutralizing antibody or genetic depletion of the receptor significantly counteracted motor neuron loss (Figure 2B). Since TNFα can induce TNFR2 expression in astrocytes, the authors postulated that high levels of mTNFα in neurons may prompt its interaction with astrocytes, triggering TNFR2 over-expression and generating an auto-regulatory loop that is involved in neuronal cell death. The deleterious role of the receptor was partially confirmed in vivo in SOD1^G93A^ mice. Briefly, the lack of TNFR2 resulted in partial, but significant, preservation of lumbar motor neurons, axons in sciatic nerves, neuromuscular junctions and tibialis muscle fibers at the endstage. Besides this, it was also identified reduced astrocyte reactivity in the lumbar spinal cord. However, these positive effects did not correlate with any improvements in motor performance or survival [122].

On the other hand, our groups demonstrated that TNFR1 mediates neuroprotective effects, promoting the expression of the trophic factor GDNF. Initially, we found that the stimulation of astroglial TNFR1 by exogenous TNFα could induce both the expression and secretion of GDNF by spinal cord astrocytes in vitro as well as its production within the CNS in vivo. In both cases, the deletion of TNFR1 abolished GDNF rises, an effect that was not compensated by the preservation of TNFR2 (Figure 2C). Next, we confirmed a strict correlation in the expression of the TNFα-TNFR1-GDNF protein triad in spinal cord from ALS patients and SOD1^G93A^ mice. Conversely, the disruption of TNFR1 signaling affected GDNF levels and significantly accelerated lumbar motor neuron degeneration and disease progression in SOD1^G93A^;tnfr1^−/−^ double mutant mice. Furthermore, the ablation of TNFR1 reduced the number of reactive astrocytes, compromising their trophic and supportive functions toward motor neurons. In view of these findings, we hypothesized that the progressive increase in the expression of TNFα detected in SOD1^G93A^ spinal cords at the onset and symptomatic stage of the disease may be a positive reaction of the affected tissues to limit the loss of motor neurons through the stimulation of TNFR1, with consequent production of astrocytic GDNF. It is to mention that GDNF might act not only in a paracrine way on neighboring motor neurons, but also in an autocrine manner by preventing the degeneration of astrocytes themselves [52,123]. Our data suggest that activation of the identified astrocytic TNFα-TNFR1-GDNF pro-survival pathway at the time of disease onset is insufficient to revert the irreversible process of motor neuron cell death, muscle denervation and atrophy. To exert neuroprotective effects, it must be activated within a specific time-window, in which motor neurons are injured but are still alive [62].

## 5. Concluding Remarks

Neuroinflammation is a prominent pathological feature of ALS, a fatal disorder caused by degeneration of motor neurons. For decades, the neuroinflammatory reaction has been considered a secondary effect of the neurodegenerative process. Recent evidence has, however, challenged this view and revealed that this phenomenon is involved in the pathophysiology of this disease. During the neuroinflammatory response, glial cells become reactive, undergoing a series of morphological, biochemical and molecular modifications, associated with an increased production of many factors, including the pro-inflammatory cytokine TNFα. Several studies have demonstrated a progressive up-regulation of TNFα and its receptors in both ALS patients and animal models, from the pre-symptomatic phase until the endstage of the disease, suggesting a key role for the TNFα/TNFR system in the pathogenesis and progression of ALS. Yet, earlier investigations aiming to understand the role of this cytokine in ALS gave apparently discrepant results. Both beneficial and detrimental effects of the cytokine were reported. These opposite activities of TNFα may be triggered by multiple factors, such as levels and duration of TNFα expression, type of receptor activated, as well as the state of the surrounding microenvironment.

Among the detrimental actions triggered by TNFα, it was reported its capacity to exacerbate glutamate-mediated excitotoxicity, to contribute to pharmacoresistance, and to induce motor neuron loss through the activation of TNFR2. This amount of evidence encouraged the development of anti-TNFα agents. Yet, generic anti-inflammatory treatments determined only partial improvements in the disease manifestations. For example, the administration of minocycline and thalidomide two TNFα synthesis inhibitors, modestly improved survival in ALS mouse models, but they did not effectively modulate disease progression in patients (ClinicalTrials.gov Identifier: NCT00047723—Study Start Date: 2003; NCT00140452). These negative outcomes may reflect the fact that patients, unlike mice, are usually treated at advanced stages of disease progression when motor neurons are already significantly damaged. To date, no other clinical trial has been conducted in the context of ALS using TNFα antagonist. However, a wide range of anti-TNFα molecules (e.g., infliximab, adalimumab, etanercept, lenercept) have been developed and used for the treatment of both inflammatory conditions, such as rheumatoid arthritis, ankylosing spondylitis and Crohn’s disease, as well as neurodegenerative disorders, e.g., multiple sclerosis (MS) and Alzheimer’s disease (AD) [80]. These treatments are associated with serious adverse effects, probably due to the non-specific activity of TNFα inhibitors. In 2011 Brambilla et al. tested, in a model of MS, the XPro1595 antagonist, which selectively inhibits soluble TNF without affecting transmembrane TNF, comparing its effects to those of the non-selective TNF inhibitor etanercept. They found that mice treated with XPro1595 recovered from paralysis and displayed significant axonal preservation and remyelination, compared to animals receiving etanercept [124]. Based on these promising preclinical results, XPro1595 has recently entered in a multicenter, phase 1b open-label clinical trial to determine its safety, tolerability and efficacy in patients affected by mild to moderate AD (ClinicalTrials.gov Identifier: NCT03943264—Study Start Date: 2019). Importantly, XPro1595 will be soon tested also in ALS patients.

At variance with this line of thought, Petitpain and collaborators recently analyzed the relationship between TNFα inhibitor exposure in patients with inflammatory disorders and ALS development. They concluded that, although a direct causal relationship must be interpreted carefully, TNF antagonism could act as a predisposing or risk factor for ALS [125].

This latter hypothesis supports our recent findings that imply a neuroprotective effect of TNFα, through the activation of astroglial TNFR1 and the consequent production of GDNF, and suggests that inhibition of the TNFα/TNFR system may interfere with this pro-survival pathway, thereby resulting harmful for motor neurons. Thus, it is reasonable to hypothesize that controlled administration of modified forms of TNFα, specifically targeting astroglial TNFR1 during the early phase of the disease, might exert a potential therapeutic role in the context of ALS. Yet, further investigations and a better understanding of the multiple TNFα functions in ALS are needed to enable the development of effective therapies designed to strengthen or suppress the protective and detrimental roles of the cytokine, respectively.

## Data Availability

Not applicable.
